# Meteorological Table

**Published:** 1813-10

**Authors:** 


### METEOROLOGICAL TABLE.

### *From August 25, to September 25,* 1813.

| | D. | Therm. | | | Barom. | | Hygrom. Dry. | | | —Damp. | Winds. | Atmos. Variation. |
|---|---|---|---|---|---|---|---|---|---|---|---|---|
| ● | 26 | 61 | 66 | 61 | $30^{3}$ | 2 | 22 | 35 | 30 | | N. | F... — ....C. |
| | 27 | 61 | 65 | 60 | $30^{2}$ | — | 21 | 26 | 22 | | NE..N. | F.. C.. F.. C... |
| | 28 | 58 | 63 | 58 | $30^{2}$ | — | 22 | 28 | 19 | | NE.. | C.. —... R.. |
| | 29 | 60 | 63 | 59 | $30^{2}$ | — | 14 | 10 | 12 | | E.SE.N. | F.. R.. F.... |
| | 30 | 58 | 65 | 60 | $30^{2}$ | — | 14 | 26 | 24 | | NE.NW. | C.... F....— ... |
| | 31 | 62 | 66 | 62 | $30^{3}$ | 2 | 15 | 20 | 15 | | NE E. | F.. — ... —.... |
| | 1 | 62 | 66 | 62 | 30 | $29^{3}$ | 14 | 10 | — | | S.E.. | C.. R.. — ... |
| ◐ | 2 | 62 | 64 | 59 | $29^{7}$ | 8 | 8 | — | — | | SW. | F... R. F.... |
| | 3 | 59 | 66 | 62 | $29^{9}$ | — | 6 | 22 | 11 | | SE. | F.. R. F... — .. |
| | 4 | 63 | 54 | 61 | $29^{7}$ | — | 5 | 2 | 4 | | SW... | C...R...F.— R..inN |
| | 5 | 60 | 64 | 60 | $29^{6}$ | — | — | 15 | — | 1 — — | SW.. | R....F..C..R.. in N |
| | 6 | 59 | 60 | 55 | $29^{2}$ | 4 | 4 | 20 | 15 | | W... | R.... C.. F.... |
| | 7 | 55 | 60 | 53 | $29^{4}$ | 6 | 15 | — | 16 | | W.. | F... R. F.... |
| | 8 | 49 | 53 | 50 | $29^{6}$ | 7 | 13 | 18 | 13 | | NW... | F.... R.. C.. F.. |
| | 9 | 47 | 56 | 53 | $29^{7}$ | $30^{1}$ | 11 | 20 | 13 | | NW.. | F.... — ... — .... |
| ○ | 10 | 51 | 60 | 57 | $30^{2}$ | 3 | 11 | 20 | 18 | | N.. | F... — ... — .... |
| | 11 | 59 | 65 | 60 | $30^{2}$ | — | 16 | 20 | 12 | | SW... | F... — ... —.... |
| | 12 | 61 | 67 | 61 | $30^{1}$ | $26^{9}$ | 9 | 25 | 15 | | SW... | F.. — .. — .. |
| | 13 | 58 | 65 | 60 | 30 | — | 25 | 35 | 16 | | NW.. | F...—...C..R... inN. |
| | 14 | 56 | 64 | 60 | 30 | 2 | 15 | 30 | 25 | | NW.. | F.. — ... — ... |
| | 15 | 55 | 63 | 60 | $30^{2}$ | — | 16 | 20 | 15 | | SW.. | F... — ... — ... |
| | 16 | 60 | 64 | 62 | $30^{2}$ | 3 | 6 | 4 | 3 | | W.. | F... R. C.. F.. |
| ◑ | 17 | 61 | 66 | 62 | $30^{3}$ | — | 6 | 13 | 10 | | NW.. | F... — ... C.. |
| | 18 | 61 | 65 | 61 | $30^{3}$ | 1 | 10 | 16 | 12 | | E.. | F... C.. F.. C. |
| | 19 | 59 | 65 | 60 | 30 | — | 9 | 25 | 9 | | SE.. | F...— .... — .. |
| | 20 | 58 | 65 | 59 | 30 | — | 10 | 21 | 12 | | E.. | F... — .... — .... |
| | 21 | 56 | 64 | 60 | 30 | — | 11 | 19 | 11 | | E.. | F.... C.. F.. |
| | 22 | 57 | 65 | 60 | 30 | — | 10 | 15 | 9 | | N..NE. | F...—.. R.— .. F... |
| | 23 | 58 | 62 | 59 | $30^{1}$ | 30 | 8 | 9 | 10 | | NE.. | F... R.. — ... — .. |
| ● | 24 | 60 | 64 | 57 | $30^{1}$ | 2 | 17 | — | 11 | | NE.. | F... C.. F.. |
| | 25 | 58 | 61 | 57 | $30^{3}$ | 2 | 15 | — | 11 | | NE.. | F... C... R. |

Quantity of rain from the 26th of August to the 25th of September, one inch $\frac{12}{100}$.

The general character of the weather in this interval has been as favorable for the harvest as in the preceding; and, though a greater quantity of rain has fallen, it has not been in a degree to impede the farmer in this important operation. The range of the mercury has been remarkably great, from $29^{2}$ on the morning of the 6th, when the heaviest fall of rain that had happened for many weeks occurred, to $30^{3}$. This great and rapid fall of the mercury in the barometer, was preceded, on the 4th, by sudden stormy gusts of wind from the S.W. The degree of temperature has been sometimes below the usual standard of the season; on the 8th the thermometer was down to 45 at six in the morning, and probably, in some situations exposed from N.W. which was then blowing, to a strong wind some degrees lower.

Cholera seems entirely to have vanished in this interval, and a tendency to catarrhal affections, and rheumatism, to have arisen. Rubeola still is frequent among children.

*Prince's Street, Cavendish Square, Sept.* 26.

MONTHLY

